# Effects of Mean Artery Pressure and Blood pH on Survival Rate of Patients with Acute Kidney Injury Combined with Acute Hypoxic Respiratory Failure: A Retrospective Study

**DOI:** 10.3390/medicina57111243

**Published:** 2021-11-14

**Authors:** Chi-Hua Ko, Ying-Wei Lan, Ying-Chou Chen, Tien-Tsai Cheng, Shan-Fu Yu, Abdulkadir Cidem, Yu-Hsien Liu, Chia-Wen Kuo, Chih-Ching Yen, Wei Chen, Chuan-Mu Chen

**Affiliations:** 1 Department of Life Sciences, and Ph.D. Program in Translational Medicine, National Chung Hsing University, Taichung 402, Taiwan; kochbye@gmail.com (C.-H.K.); bublelanwilliam@gmail.com (Y.-W.L.); cidema.kadir@gmail.com (A.C.); yuhsien000@yahoo.com.tw (Y.-H.L.); kuochiawen@yahoo.com.tw (C.-W.K.); 2 Division of Rheumatology, Allergy and Immunology, Chang Gung Memorial Hospital, Yunlin 638, Taiwan; 3 Division of Rheumatology, Allergy and Immunology, Chang Gung Memorial Hospital, Kaohsiung 833, Taiwan; r820713@cgmh.org.tw (Y.-C.C.); tiantsai0919@gmail.com (T.-T.C.); yu820@cgmh.org.tw (S.-F.Y.); 4 Department of Molecular Biology and Genetics, Erzurum Technical University, Erzurum 25250, Turkey; 5 Department of Nephrology, Jen-Ai Hospital, Dali, Taichung 412, Taiwan; 6 Department of Internal Medicine, Taichung Armed Forces General Hospital, Taichung 411, Taiwan; 7 Department of Internal Medicine, China Medical University Hospital, and College of Health Care, China Medical University, Taichung 404, Taiwan; d5210@mail.cmuh.org.tw; 8 Division of Pulmonary and Critical Care Medicine, Chia-Yi Christian Hospital, Chiayi 600, Taiwan; peteralfa2004@gmail.com; 9 The iEGG and Animal Biotechnology Center, and the Rong Hsing Research Center for Translational Medicine, National Chung Hsing University, Taichung 402, Taiwan

**Keywords:** acute kidney injury (AKI), acute hypoxic respiratory failure (AHRF), mean arterial pressure (MAP), blood pH, lactate

## Abstract

*Background and Objectives:* In the intensive care unit (ICU), renal failure and respiratory failure are two of the most common organ failures in patients with systemic inflammatory response syndrome (SIRS). These clinical symptoms usually result from sepsis, trauma, hypermetabolism or shock. If this syndrome is caused by septic shock, the Surviving Sepsis Campaign Bundle suggests that vasopressin be given to maintain mean arterial pressure (MAP) > 65 mmHg if the patient is hypotensive after fluid resuscitation. Nevertheless, it is important to note that some studies found an effect of various mean arterial pressures on organ function; for example, a MAP of less than 75 mmHg was associated with the risk of acute kidney injury (AKI). However, no published study has evaluated the risk factors of mortality in the subgroup of acute kidney injury with respiratory failure, and little is known of the impact of general risk factors that may increase the mortality rate. *Materials and Methods:* The objective of this study was to determine the risk factors that might directly affect survival in critically ill patients with multiple organ failure in this subgroup. We retrospectively constructed a cohort study of patients who were admitted to the ICUs, including medical, surgical, and neurological, over 24 months (2015.1 to 2016.12) at Chiayi Chang Gung Memorial Hospital. We only considered patients who met the criteria of acute renal injury according to the Acute Kidney Injury Network (AKIN) and were undergoing mechanical ventilator support due to acute respiratory failure at admission. *Results:* Data showed that the overall ICU and hospital mortality rate was 63.5%. The most common cause of ICU admission in this cohort study was cardiovascular disease (31.7%) followed by respiratory disease (28.6%). Most patients (73%) suffered sepsis during their ICU admission and the mean length of hospital stay was 24.32 ± 25.73 days. In general, the factors independently associated with in-hospital mortality were lactate > 51.8 mg/dL, MAP ≤ 77.16 mmHg, and pH ≤ 7.22. The risk of in-patient mortality was analyzed using a multivariable Cox regression survival model. Adjusting for other covariates, MAP ≤ 77.16 mmHg was associated with higher probability of in-hospital death [OR = 3.06 (1.374–6.853), *p* = 0.006]. The other independent outcome predictor of mortality was pH ≤ 7.22 [OR = 2.40 (1.122–5.147), *p* = 0.024]. Kaplan-Meier survival curves were calculated and the log rank statistic was highly significant. *Conclusions:* Acute kidney injury combined with respiratory failure is associated with high mortality. High mean arterial pressure and normal blood pH might improve these outcomes. Therefore, the acid–base status and MAP should be considered when attempting to predict outcome. Moreover, the blood pressure targets for acute kidney injury in critical care should not be similar to those recommended for the general population and might prevent mortality.

## 1. Introduction

In intensive care units (ICUs), multiple organ failure is associated with increased mortality and prolonged hospital stay, and renal failure and respiratory failure are the two most common organ failures in critically ill patients [1,2]. There are many recognized risk factors associated with death in ICU with renal failure, including old age, prolonged hospitalization, high Acute Physiology, and Chronic Health Evaluation (APACHE II) score, comorbidities, sepsis, invasive mechanical ventilation, etc. [3]; risk factors for respiratory failure, including old age, immune responses, or coagulation dysfunction have been reported to be associated with death [4].

Systemic inflammatory response syndrome results in a systemic inflammatory process arising from nonspecific insults leading to multiple organ failure [5]. When this syndrome is the result of a life-threatening organ dysfunction caused by the host’s uncontrollable response to a confirmed infectious pathogen, it is termed sepsis. Organ dysfunction is defined as a ≥2-point change in the total Sequential Organ Failure Assessment (SOFA) score after the patient is determined to be infected [6,7]. In sepsis, systemic inflammatory response results in cytokine release, which changes the patient’s blood pressure and organ perfusion [8,9]. Mounting evidence has indicated that the body can tolerate hypotension in this situation and that a high blood pressure target does not result in significant differences in mortality [5,10]. Therefore, it is clearly stated in the sepsis guidelines that it is only necessary to maintain the mean arterial pressure (MAP) above 65 mmHg [11].

Sepsis-induced acute kidney injury (AKI) is well-recognized for its impact on the outcomes of patients admitted to the ICU [12]. Respiratory failure in patients with AKI is a devastating consequence that greatly increases patient mortality to higher than 80%. Diminished renal function is accompanied by alterations in homeostasis of the volume status, which can affect pulmonary function—for example, by increasing respiratory workload and decreasing lung compliance. Respiratory failure in patients with AKI is a life-threatening complication of pulmonary edema [13]. This close connection between the lungs and the kidneys is important in patients with multiple organ failure.

Hypotension is a dangerous signal in ICUs. It can cause insufficient perfusion, increased hospital stay, or death. The regulation of blood pressure is often an important issue in ICUs [14]. Therefore, there are still different opinions on the blood pressure that patients with renal failure need to maintain when receiving intensive care. Studies have shown that in patients with chronic renal failure, their blood pressure is originally higher than average [15].

Additionally, mounting evidence showed that patients in the ICU can tolerate low MAP without worsening the prognosis or worsening kidney function [16,17]. However, if the same low MAP standard is used, it will worsen the renal function in patients with renal failure [18], and a relatively high MAP can prevent the occurrence of acute renal failure. In patients with renal failure, reduced salt excretion or excessive body water can cause chronic hypertension [19]. In these patients, we find that their blood pressure is very difficult to control. However, when these patients encounter an acute renal function change, we find patients’ urine output will decrease [20,21]. Many studies have also pointed out that maintaining a high MAP in these patients is actually harmless [22].

The development of kidney injury is a risk factor for the development of respiratory failure and chronic hypertension. Preserving perfusion pressure for optimizing flow in various organs is very important. This target is controversial, because a study showed that patients with chronic hypertension target high MAP, resulting in increased organ perfusion, but found no evidence of improved survival rate [23]. There is no study that has evaluated the risk factors for mortality in the subgroup of AKI with respiratory failure, and little is known regarding the general risk factors that may increase the mortality rate.

In this study, we aimed to determine the risk factors that might directly affect survival in critically ill patients with combined AKI and respiratory failure. We compared the prognosis in surviving and non-surviving patients and examined the effect of hemodynamic variables and acidosis on outcomes in this subgroup. Knowledge of such general determinants of outcome in critically ill patients with AKI and respiratory failure not only help improve prognostic evaluation, but also help indicate what therapy should be administered; accordingly, research should be conducted to improve both short-term and long-term outcomes.

## 2. Materials and Methods

### 2.1. Study Populations

**Figure 1:**
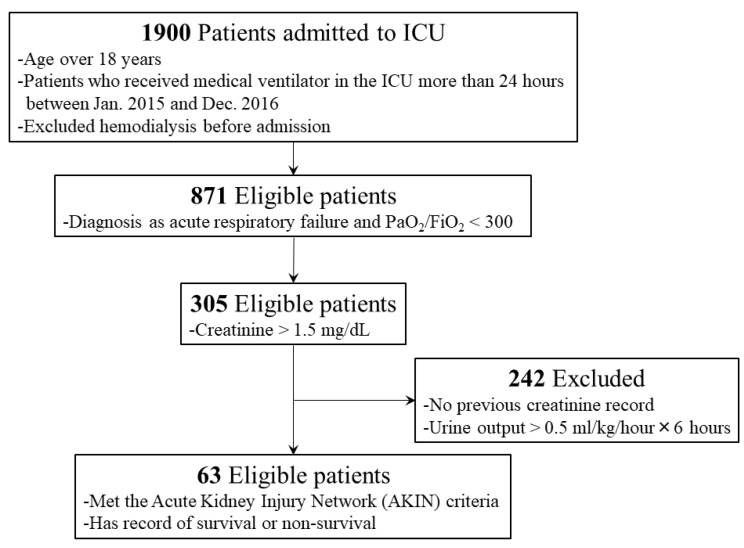
Inclusion and exclusion criteria for ICU patient enrollment in this retrospective study.

We retrospectively constructed a cohort study of patients who were admitted to the ICUs, including medical, surgical and neurological, over 24 months (January 2015 to December 2016) at Chiayi Chang Gung Memorial Hospital. Patients receiving chronic hemodialysis before admission and those hospitalized less than 24 h were excluded. We only considered adult patients (age ≥ 18 years) who met the criteria of the Acute Kidney Injury Network (AKIN) and were undergoing mechanical ventilator support due to acute respiratory failure on admission (Figure 1). The study was approved by the institutional review board of Chang Gung Memorial Hospital (IRB number: 201800112B0C501).

### 2.2. Data Collection

Demographic data were retrospectively retrieved from the electronic hospital database and laboratory data were retrospectively retrieved from the laboratory data base. After merging data from various sources, we performed manual data verification. The final data included demographics, laboratory data, physiological data, diagnosis, and hospital outcome. All the laboratory data were collected during the first 24 h of ICU admission. The severity of illness was calculated as the sequential organ failure assessment score (SOFA), based on the worst variables recorded during the first 24 h of admission to the ICU. The patient’s admission diagnosis is based on the main ICD code of the hospitalization. Cardiac output is calculated using the estimated value in the patient’s past or present ultrasound report. Our blood pressure measurement uses an automatic physiological monitor to collect multiple records of the patient’s first day of blood pressure and average them.

### 2.3. Acute Kidney Injury Characterization

Acute kidney injury (AKI) is generally defined as an abrupt and sustained decrease in kidney function. Patients who met the criteria of Acute Kidney Injury Network (AKIN) were classified as having AKI [20]. The diagnostic criteria of AKI were defined as a maximal change in serum creatinine more than 0.3 mg/dL, a percentage increase in serum creatinine of more than 50% (1.5-fold from baseline), or a reduction in urine output (less than 0.5 mL/kg per hour for more than 6 h) after optimal hydration during the first 48 h at ICU admission.

Patients were stratified according the AKIN stage: stage 1 = increase in serum creatinine greater than or equal to 0.3 mg/dL, increase to more than or equal to 150% to 200% of baseline, or urine output less than 0.5 mL/kg per hour for more than 6 h; stage 2 = increase in serum creatinine to greater than 200% to 300% of baseline, or urine output less than 0.5 mL/kg per hour for more than 12 h; and stage 3 = increase in serum creatinine to more than 300% of baseline, serum creatinine greater than or equal to 4.0 mg/dL with an acute increase of at least 0.5 mg/dL, urine output less than 0.3 mL/kg per hour for 24 h, or anuria for 12 h. The reference serum creatinine was the lowest creatinine value recorded within 3 months of the event. If a reference serum creatinine value is not available within 3 months and AKI is suspected, we repeated serum creatinine measurement within 24 h [24].

### 2.4. Respiratory Failure Characterization

Acute respiratory failure was defined as acute impairment of gas exchange between lung and blood causing hypoxia or hypercapnia. Hypoxia was defined as arterial partial pressure of oxygen less than 60 mmHg breathing room air, and hypercapnia was defined as arterial partial pressure of carbon dioxide greater than 50 mmHg breathing room air. Patients who met the acute respiratory definition required support with a mechanical ventilator to remain alive upon ICU admission [25].

### 2.5. Outcome Measurement

The primary outcome was in hospital mortality or survival, defined as survival status at the number of days from the date of admission to hospital discharge and survival status after ICU discharge.

### 2.6. Statistical Analysis

The measured values (blood pressure and arterial pH) were compared for survivors and non-survivors. Demographic data and selected laboratory values were verified for normal distribution using the Kolmogorov-Smirnov test. Continuous variables were expressed as the mean ± standard deviation compared using the Student’s two tail *t*-test when normally distributed and using the Mann-Whitney U test when not. Categorical data were expressed as frequency and percentage and were compared using the Chi-square test. Multiple Cox regression analysis was used to predict outcome variables that were independently associated with death. Significant variables were entered into a backward, stepwise, Cox regression model. Survival curves of the lactate, pH, and MAP groups were drawn using the Kaplan-Meier method, and we tested the difference between groups using the log-rank test. All tests were double-sided, and *p*-value less than 0.05 was considered statistically significant. All statistical analyses were carried out using SPSS version 22.0 for Windows (SPSS Inc., Chicago, IL, USA).

## 3. Results

### 3.1. Patient Characteristics

During the study period, our study involved 63 critically ill patients with confirmed diagnoses of AKI under ventilator support, including 46 males and 17 females. The baseline characteristics of the patient cohort, including demographic data, SOFA score and major diagnosis at admission are presented according to survival and non-survival in Table 1. The mean age was 66.63 ± 16.40 and the mean admission SOFA score was 11.06 ± 2.16. There were no significant differences in terms of age, gender, and SOFA score between survivors and non-survivors. The overall mortality rate was 63.5%, and the non-survival patients experienced longer hospital stays (35.3 ± 23.45 days). The major diagnosis at ICU admission in this cohort was cardiovascular disease (31.7%) followed by respiratory disease (28.6%). In the surviving group, the main diagnosis of hospital admission was heart disease, while in the non-surviving group, the respiratory disease accounted for the majority. All patients developed AKI during the first week after admission, including 25 (39.7%) fulfilling the criteria for AKIN stage 1, 15 (23.8%) fulfilling the criteria for AKIN stage 2, and 23 (36.5%) fulfilling the criteria for AKIN stage 3. Twenty-five participants required dialysis therapy, including 13 (20.6%) undergoing hemodialysis and 12 (19%) undergoing hemofiltration.

### 3.2. Basic Physiological and Hemodynamic Data

Table 2 lists basic data, blood gas analysis, hemodynamic data, and pulmonary characteristics at admission in both groups. The mean serum creatinine concentration before onset of disease was 2.42 ± 2.16 mg/dL for all AKI patients. At admission, only MAP (mean arterial pressure), WBC (white blood cell), pH (acid–base balance), and lactate were significantly different for survivors and non-survivors. The MAP of the non-survival group was 74.78 ± 13.48 mmHg, and the mean pH was 7.25 ± 0.11 significantly lower than that of the survival group. The mean WBC of the non-survival group was 17.74 ± 8.15 (10^9^/L), and the mean lactate was 68.66 ± 46.15, significantly higher than that of the survival group. The non-survival group exhibited a higher mean positive fluid balance than did the survival group; however, the difference was insignificant.

### 3.3. Receiver-Operating Characteristic (ROC) Curve

**Figure 2:**
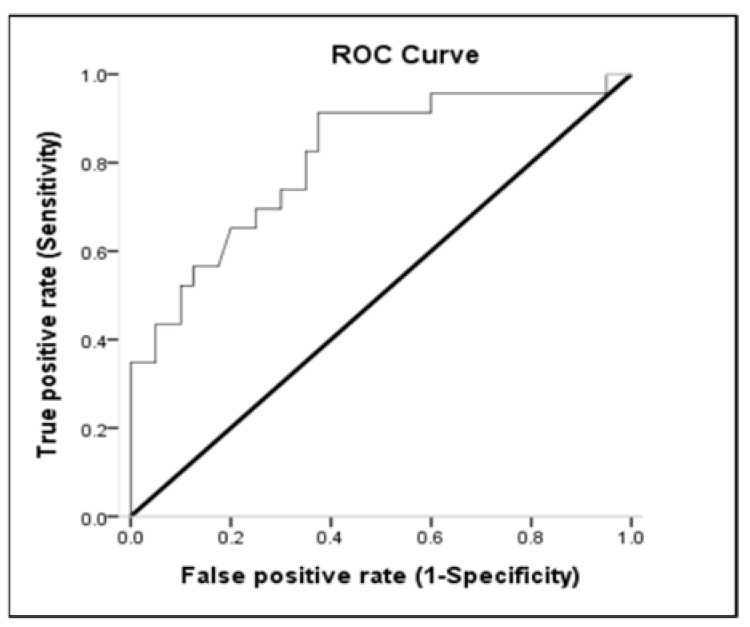
The receiver-operating characteristic (ROC) curve of ability of lactate to predict overall mortality.

**Figure 3:**
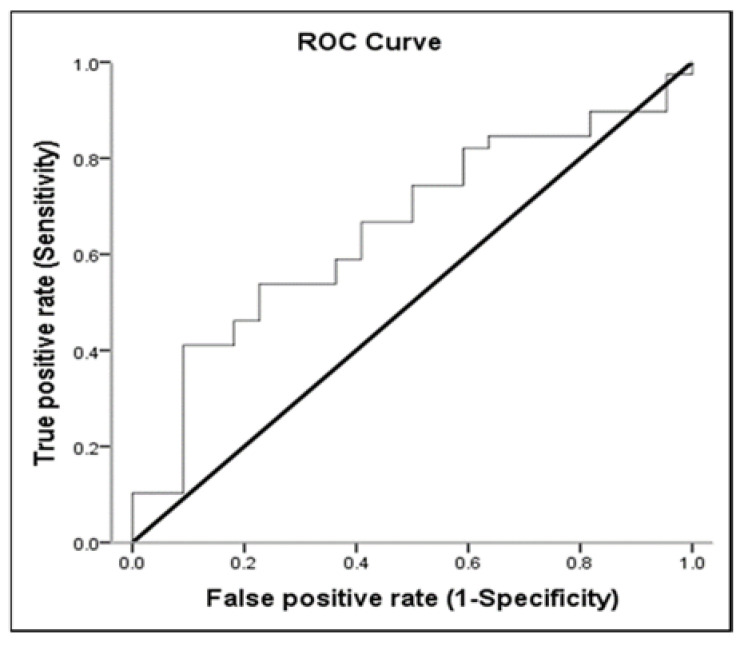
The receiver-operating characteristic (ROC) curve of ability of MAP to predict overall mortality.

**Figure 4:**
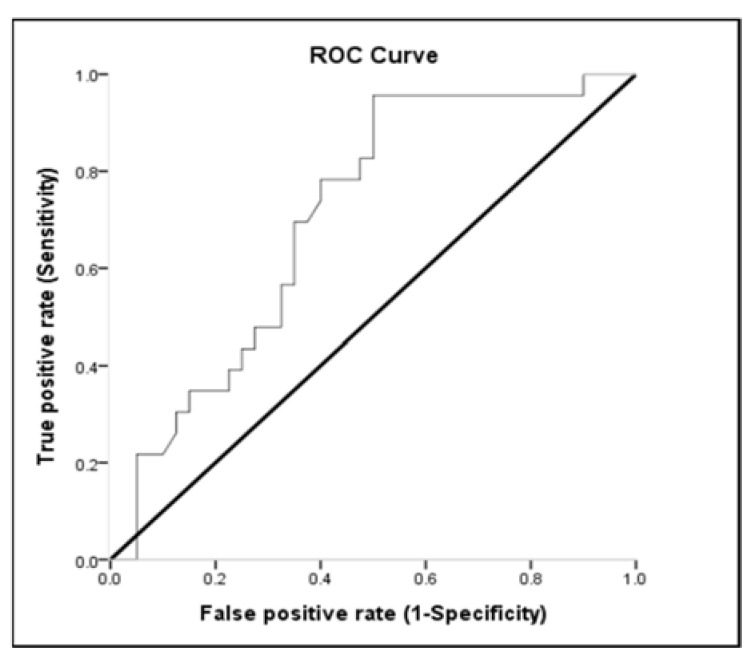
The receiver-operating characteristic (ROC) curve of ability of pH to predict overall mortality.

Lactate, MAP, and pH discriminated survivors from non-survivors. The area under the ROC curve of lactate was 0.654 (95% CI: 0.513–0.795; *p* < 0.05) for mortality (Table 3 and Figure 2). Sensitivity and specificity for overall deaths of lactate > 51.8 were 53.85% and 77.27%, respectively. The positive predictive value (PPV) and negative predictive value (NPV) of lactate for mortality were 80.77% and 48.57%, respectively (Table 4). The area under the ROC curve of MAP was 0.810 (95% CI: 0.694–0.926; *p* < 0.001) for overall mortality (Table 3 and Figure 3). Sensitivity and specificity for overall death of MAP ≤ 77.16 were 62.50% and 91.30%, respectively. The PPV and NPV values of MAP for mortality were 92.59% and 58.33%, respectively (Table 4). The area under the ROC curve of pH was 0.693 (95% CI: 0.560–0.826; *p* = 0.013) for overall mortality (Table 3 and Figure 4). Sensitivity and specificity for overall death of pH ≤ 7.22 were 47.50% and 95.65%, respectively. The PPV and NPV values of pH for mortality were 95.00% and 51.16%, respectively (Table 4). The area under the ROC curve of WBC was 0.612 (95% CI: 0.468–0.757; *p* = 0.147) for overall mortality; however, this result was not significant.

### 3.4. ICU Outcome Analysis

**Figure 5:**
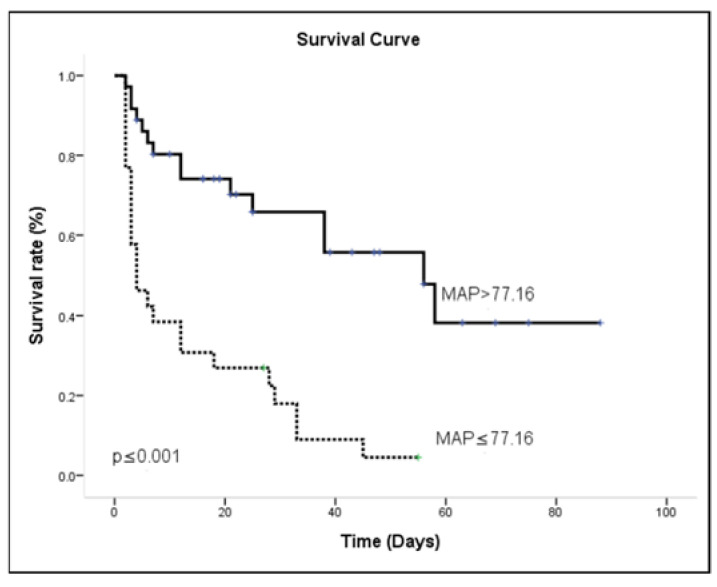
Survival curve using the Kaplan-Meier method for MAP.

**Figure 6:**
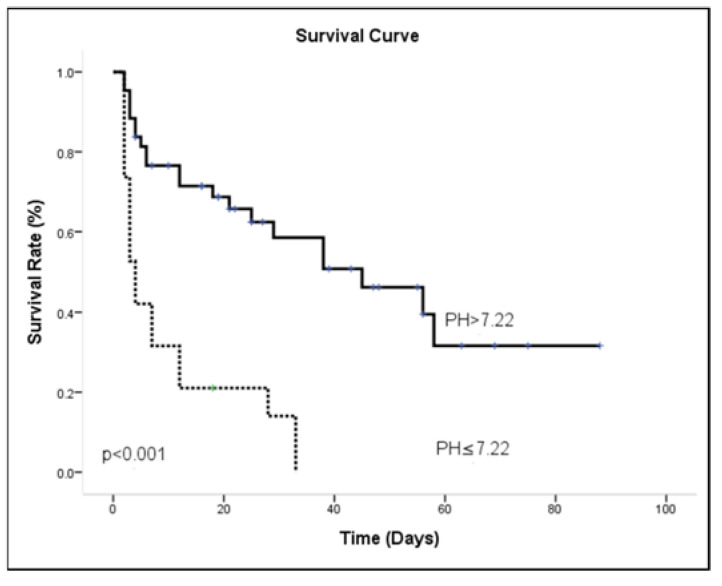
Survival curves using the Kaplan-Meier method for pH.

Most patients (73%) had suffered sepsis during their ICU admission, and the mean length of hospital stay was 24.32 ± 25.73 days. The survivors had significantly more cardiologic disease at admission then did non-survivors. In general, lactate > 51.8 mg/dL, MAP ≤ 77.16 mmHg and pH ≤ 7.22 were associated with mortality in univariate analysis. The risk of in-patient mortality during AKI combined with respiratory failure is shown in Table 5, reporting multivariable Cox regression survival model results. Adjusting for other covariates, patients with MAP ≤ 77.16 mmHg were associated with a higher probability of hospital death [OR = 3.06 (1.374–6.853); *p* = 0.006]. The other independent outcome predictor was pH ≤ 7.22 with a higher risk of hospital death [OR = 2.40 (1.122–5.147); *p* = 0.024]. Kaplan-Meier survival curves were calculated using the MAP ≤ 77.16 (Figure 5) and pH ≤ 7.22 (Figure 6). The results for the log-rank statistics were highly significant.

## 4. Discussion

The main findings in our study were that (a) the ICU mortality rate in acute kidney injury combined with respiratory failure were higher than reported in several other studies [1,2,26]; and (b) the two most important risk factors for death in the ICU were hypotension and acidosis.

In Taiwan, a previous study reported that the ICU mortality of adult patients with an ICU stay of 48 h or more was 22.5% [27]. The ICU mortality in other counties was approximately 17–25% reported from the USA, Canada, and Japan [28]. Nevertheless, mortality in our study was 63.5% higher than previously reported in the general population because our patients were in critical condition with acute kidney injury. Among the patients with AKI, the percentage of deaths in our study was generally similar to those reported in the literature, with a mortality rate of 66% [29]. Moreover, in previous studies, the mortality rate of patients with AKI was high, exceeding 60% when only ICU patients are analyzed [30,31,32,33].

Some factors inducing renal failure are responsible for the increased mortality rate. However, Santos and coworkers reported mortality rates in patients with ischemic (66%) and mixed (63%) acute renal failure to be almost twice the rate of patients with nephrotoxic acute renal failure (38%) [30,34]. As a result, ischemia-related renal failure might be the determining factor in mortality rates in patients with acute renal failure. Several studies also have demonstrated the impact of ischemic factors (hypotension, shock and sepsis) on mortality rates in patients with acute renal failure [31,35]. Our study provides new evidence of the importance of hypotension (MAP < 77 mmHg) as a risk factor for mortality in patients with AKI. This result was similar to that of Wang et al. [36] who found predialysis MAP < 90 mmHg and MAP that decreased during dialysis were associated with increased risk of death in chronic hemodialysis patients.

In patients undergoing hemodialysis or those with chronic hypertension, a number of observational cohort studies have reported U-shaped or inverted-J relationships between conventional blood pressure measures (systolic, diastolic, and mean arterial) and mortality. These studies reported high prevalence of hypertension in the end-stage renal disease (ESRD) population [37,38]. Therefore, it is important to note that the effect of various mean arterial pressures on renal function, e.g., mean arterial pressure less than 75 mmHg, was associated with the development of acute kidney injury [22].

Sepsis is another factor classically associated with death in ICU patients. In the current study, sepsis occurred in 73% of the patients, similar to those previously reported by other investigators (64.4%) [29]. Recent data underline the strong prognostic impact of hypotension and cardiovascular failure in critically ill patients with sepsis [39]. Most patients with sepsis showed some degree of myocardial depression. Despite a compensatory increase in CO, the elevated SVR, hypovolemia, and myocardial depression induce hypotension, otherwise known as septic shock. Without aggressive fluid resuscitation in this phase, profound hypotension and progressive acidosis develop, leading to irreversible shock, multiple organ failure and death. The Surviving Sepsis Campaign Bundle states that vasopressin should be given to the patient to maintain a mean arterial pressure > 65 mmHg if the patient is hypotensive after fluid resuscitation, blood should be obtained for measuring lactate and blood cultures, and fluids and antibiotics should be administrated [40]. Nevertheless, increasing the MAP from 65 to 85 mmHg with nor-epinephrine neither affects metabolic variables nor improves renal function in septic shock [16,40]. A higher MAP (approximately 75 to 85 mmHg) may be preferable without harm in patients with chronic arterial hypertension, including patients with renal failure [40]. In the subgroup with acute or chronic renal failure, chronic hypertension is ubiquitous, and it has long been thought that renal disease decreases salt excretion, leading to volume overload and consequent hypertension [15]. In our study, the area under the ROC curve of MAP was 0.810 (95% CI: 0.694–0.926; *p* < 0.001) for overall mortality. Sensitivity and specificity for overall death of MAP ≤ 77.16 were 62.50% and 91.30%, respectively. This means that MAP > 77.16 mmHg is a good indicator to distinguish whether patients with renal failure combined with respiratory failure will survive. Therefore, in our study, MAP ≤ 77.16 mmHg was associated with higher probability of hospital death [OR = 3.06 (1.374–6.853); *p* = 0.006]; therefore, the blood pressure targets for renal failure in critical care should not be similar to that recommended for the general population.

The pH level of the survival group was significantly higher than that of the non-survival group. In the ICU, metabolic acidosis is the most frequent acid–base disorder and hyperlactatemia may be the cause of metabolic acidosis [41]. However, lactic acidosis, not hyperlactatemia, was found to predict hospital mortality more precisely in severe sepsis and septic shock patients [42]. Low lactate clearance in severely ill septic patients predicted poor outcome [43,44]. In the present study, there was no difference in the prevalence of lactic acidosis between survival and non-survival groups. The multiple variance analysis also showed no significance in lactate > 51.8. One possible explanation for this is that blood pH, base deficit and AG may not correlate with hyperlactatemia because they can be affected by ventilator status, renal failure and other complex acid–base disorders [45]. The other explanation is that using mechanical ventilation can decrease the severity of hyperlactatemia in patients with acute respiratory failure [46].

In accordance with our results, patients with initial arterial pH levels less than 7.22 were closely related to the predictor of mortality [OR = 2.40 (1.122–5.147); *p* = 0.024]. Gourhant and his colleagues [47] reported that pH < 7.36 was the sole independent predictor in obese patients associated with ICU admission (ROC curve AUC: 0.74). Arterial pH < 7.35 at presentation is also a useful early marker for predicting adverse outcomes in acute pancreatitis (ROC curve AUC: 0.771) [48]. The exact mechanisms are that pulmonary vascular resistance dramatically increases with hypoxia, while arterial blood pH drops below 7.3 [49]. Zapol et al. [50] also reported survivors had progressive decreases of pulmonary vascular resistance with time, whereas non-survivors tended to have increased pulmonary vascular resistance. Because of the increased pulmonary vascular resistance, left and right atrial pressures also changed significantly, and reductions in mean arterial pressures were noted [51]. These studies are similar to those reported by Bourgoin et al. [16] and Lien and Chan [52] who found that MAP and arterial pH at 24 h most strongly predicted mortality in patients with renal failure.

Although fluid balance does not reach statistical significance, there is a considerable difference between the surviving group and the non-surviving group. This result also proves that in past studies, crystalloid water was used to resuscitate patients with sepsis in the early stage, and the water supply needed to be restricted in the later stage to achieve fluid balance [17]. There were several limitations in this study. First, using existing medical records without urine output data, acute kidney injury was defined according creatinine criteria of AKIN classification [53]. The second limitation is that echocardiography was not routinely performed to confirm whether low blood pressure was due to low cardiac output or to decreased peripheral resistance. Third, the information regarding antihypertensive medications was limited. Fourth, we did not have records of the serum anion gap level; therefore, we could not know the cause of metabolic acidosis. Fifth, in the surviving group, the main diagnosis of hospital admission was heart disease, while in the dead group, the respiratory disease accounted for the majority. Because we only take the main diagnostic code of the ICD code, it will cause incomplete expression of all the patients’ diseases. This also means that our study patients may have the disadvantage of unbalanced distribution. This is the shortcoming of retrospective research that requires further research in the future.

## 5. Conclusions

In this study, we found that acute kidney injury combined with respiratory failure is associated with high mortality. High mean arterial pressure and normal blood pH might have a better prognosis. Therefore, acid–base status and MAP should be considered when attempting to predict outcome. Finally, the blood pressure targets for acute kidney injury in critical care should not be similar to those recommended for the general population and might prevent mortality.

## Figures and Tables

**Table 1:** Comparison of patient characteristics between survival and non-survival groups at admission to the intensive care unit.

	All (*n* = 63)	Survival (*n* = 23)	Non-survival (*n* = 40)	*p*
Age # ([min-max])	66.63 ± 16.40 [22.6–94.7]	67.74 ± 14.64 [31.1–92.4]	66 ± 17.48 [22.6–94.7]	0.689
Gender, male/female	46/17	17/6	29/11	0.573
SOFA score ([min-max])	11.06 ± 2.16 [7–17]	11.38 ± 2.02 [7–17]	10.52 ± 2.33 [8–15]	0.133
Diagnosis on admission *				
Respiratory diseases	18 (28.5)	3 (13)	15 (37.5)	0.021
Neurological diseases	6 (9.5)	2 (8.7)	4 (10)	
Nephrological disease	3 (4.8)	1 (4.3)	2 (5)	
Infectious disease	5 (7.9)	0 (0)	5 (12.5)	
Cardiologic diseases	20 (31.7)	13 (56.5)	7 (17.5)	
Gastroenterological diseases	10 (15.9)	3 (13)	7 (17.5)	
Hematologic diseases	1 (1.6)	1 (4.3)	0 (0)	
AKIN stage				
Stage 1	25 (39.7)	10 (43.5)	15 (37.5)	0.661
Stage 2	15 (23.8)	4 (17.4)	11 (27.5)	
Stage 3	23 (36.5)	9 (39.1)	14 (35)	
Sepsis	46 (73)	14 (60.9)	32 (80)	0.141
Lactic acidosis	12 (19)	4 (17)	8 (20)	0.800
Metabolic acidosis	47 (74.6)	17 (73.9)	30 (75)	0.924
Hemodialysis	13 (20.6)	5 (21.7)	8 (20)	0.653
Hemofiltration	12 (19)	3 (13)	9 (22.5)	
Length of hospital stay *([min-max])	22 ± 21.53 [1–88]	14.15 ± 15.99 [4–88]	35.3 ± 23.45 [1–58]	0.001

Values are means ± SD or number of patients and number (percentage); * *p* value < 0.05; Abbreviation: SOFA, sequential organ failure assessment score. Age # calculated as decimal age: hospitalized date minus birth date.

**Table 2:** Patient characteristics and physiological variables at admission to the intensive care unit.

	All (*n* = 63)	Survival (*n* = 23)	Non-survival (*n* = 40)	*p*
**Basic Data**				
BUN (mg/dL)	66.12 ± 22.17 [16.90–121.00]	69.48 ± 22.13 [31.30–121.00]	64.19 ± 22.24 [16.90–102.00]	0.366
Baseline creatinine (mg/dL)	2.42 ± 2.16 [0.64–9.85]	2.66 ± 2.08 [0.64–9.85]	2.28 ± 2.22 [0.69–9.68]	0.502
Creatinine (mg/dL)	4.73 ± 2.62 [1.34–11.38]	5.30 ± 2.53 [1.34–11.10]	4.40 ± 2.65 [1.52–11.38]	0.193
Lactate(mg/dL) *	61.21 ± 42.54 [13.60–225]	48.02 ± 32.09 [15.90–139.35]	68.66 ± 46.15 [13.60–225]	0.045
Sodium (mg/dL)	139.82 ± 6.36 [126.00–154.00]	139.59 ± 5.96 [138.00–154.00]	139.95 ± 6.66 [126.00–151.00]	0.829
Potassium (mg/dL)	4.71 ± 1.02 [2.60–7.20]	4.91 ± 0.87 [4.80–6.90]	4.59 ± 1.08 [2.60–7.20]	0.228
Hb (g/dL)	10.42 ± 2.49 [6.60–16.10]	10.13 ± 2.12 [7.00–16.00]	10.57 ± 2.68 [6.60–16.10]	0.511
WBC (10^9^/L) *	16.2 ± 7.84 [3.00–35.00]	13.53 ± 6.61 [3.00–33.80]	17.74 ± 8.15 [3.70–35.00]	0.039
**Hemodynamic Data**				
MAP (mmHg) *	83.25 ± 20.93 [50.67–153.33]	97.99 ± 23.55 [52.00–153.33]	74.78 ± 13.48 [50.67–105.67]	0.001
Heart rate (beat/min)	107.43 ± 17.87 [58.60–137.20]	102.49 ± 17.67 [64.25–128.00]	110.27 ± 17.58 [58.60–137.20]	0.096
Stroke volume (mL/beat)	64.25 ± 10.81 [35.00–86.90]	63.87 ± 11.66 [39.00–86.90]	64.47 ± 10.44 [35.00–86.00]	0.835
Fluid balance (mL/day)	894.41 ± 1362.87 [−1656.0–3978.3]	581.79 ± 1323.08 [−1656.0–3752.0]	1078.78 ± 1368.99 [−1100.0–3978.3]	0.167
**Blood Gas Analysis**				
pH *	7.28 ± 0.11 [7.04–7.50]	7.33 ± 0.08 [7.12–7.45]	7.25 ± 0.11 [7.04–7.50]	0.002
PaCO_2_ (mmHg)	40.82 ± 14.93 [21.20–84.03]	39.43 ± 10.58 [24.33–63.20]	41.64 ± 17.06 [21.20–84.03]	0.531
PEtCO_2_ (mmHg)	31.92 ± 8.03 [18.50–50.00]	32.32 ± 8.23 [18.50–49.50]	31.67 ± 8.03 [21.00–50.00]	0.774
PaO_2_ (mmHg)	116.92 ± 51.79 [33.80–233.20]	110.53 ± 56.67 [33.80–233.20]	120.6 ± 49.14 [34.95–221.37]	0.462
HCO_3_ (mmol/L)	20.87 ± 7.35 [8.60–48.80]	20.18 ± 4.3 [12.70–27.00]	21.26 ± 8.66 [8.60–48.80]	0.511
BE (meq/L)	−4.79 ± 6.73 [−16.70–20.80]	−5.21 ± 4.75 [−12.67–3.20]	−4.55 ± 7.69 [−16.70–20.80]	0.708
PAaO_2_ (mmHg)	419.75 ± 146.44 [102.90–625.79]	462.42 ± 117.62 [127.40–625.79]	398.41 ± 155.89 [102.90–619.00]	0.111
**Pulmonary Characteristics #**				
Respiratory rate (breaths/min)	22.21 ± 3.96 [14.80–31.82]	21.96 ± 4.24 [14.80–29.50]	22.35 ± 3.84 [17.00–31.82]	0.711
Tidal volume (mL)	517.11 ± 78.25 [378.40–701.00]	521.79 ± 77.93 [400.14–659.25]	514.43 ± 79.29 [378.40–701.00]	0.722
Minute volume (L/min)	11.73 ± 3.43 [6.63–20.73]	11.63 ± 3.77 [7.00–20.73]	11.78 ± 3.28 [6.63–19.75]	0.870
Peak pressure (cmH_2_O)	26.76 ± 3.68 [20.00–36.00]	26.60 ± 3.87 [20.83–36.00]	26.86 ± 3.61 [20.00–36.00]	0.792
PaO_2_/FIO_2_ ratio	148.94 ± 68.98 [43.69–298.82]	132.24 ± 68.7 [48.29–298.82]	158.55 ± 68.13 [43.69–298.82]	0.146

Variable values expressed as the mean ± SD ([min-max]); * *p* value < 0.05. #, respiratory parameters were recorded under mechanical ventilation. Abbreviations: Hb, hemoglobin; WBC, white blood cells; MAP, mean arterial pressure (=[systolic blood pressure + (2 × diastolic blood pressure)]/3); pH, acid–base balance; PaCO_2_, partial pressure carbon dioxide; PEtCO_2_, end-tidal carbon dioxide; PaO_2_, partial pressure oxygen; HCO_3_, bicarbonate; BE, base excess/base deficit; PAaO_2_, alveolar-arterial partial pressure of oxygen (PO_2_) difference; FiO_2_, fraction of inspired oxygen.

**Table 3:** Area under the ROC curve, with standard error and 95% confidence interval.

Variables	Area under the Curve (%)	(95% Confidence Interval)	*p*
Lactate ^a^	0.654	0.513–0.795	0.047
WBC ^a^	0.612	0.468–0.757	0.147
MAP ^b^	0.810	0.694–0.926	0.000
pH ^b^	0.693	0.560–0.826	0.013

^a^ Predictor for mortality; ^b^ Predictor for survival; Abbreviations: WBC, white blood cells; MAP, mean arterial pressure; pH, acid–base balance.

**Table 4:** Determination of risk assessment in acute respiratory failure with acute kidney injury according to lactate, MAP, and pH.

Factors	Sensitivity (%)	Specificity (%)	NPV (%)	PPV (%)
Lactate > 51.8	53.85	77.27	48.57	80.77
MAP ≤ 77.16	62.50	91.30	58.33	92.59
pH ≤ 7.22	47.50	95.65	51.16	95.00

Abbreviations: MAP, mean arterial pressure; pH, acid–base balance; NPV, negative predictive value; PPV, positive predictive value.

**Table 5:** Results of Cox multivariate proportional model.

Variables	Multivariate Analysis			
	Coefficient, Mean (SE)	Odds Ratio	(95% Confidence Interval)	*p*
pH ≤ 7.22	0.877 (0.389)	2.403	1.122–5.147	0.024
MAP ≤ 77.16	1.121 (0.410)	3.068	1.374–6.853	0.006

## Data Availability

Not applicable.
